# Experimental evidence of parasite-induced behavioural alterations modulated by food availability in wild capuchin monkeys

**DOI:** 10.1038/s41598-023-30262-2

**Published:** 2023-02-22

**Authors:** Ilaria Agostini, Ezequiel Vanderhoeven, Romina Pfoh, Barbara Tiddi, Pablo M. Beldomenico

**Affiliations:** 1 Parque Nacional Nahuel Huapi (CENAC-APN), Consejo Nacional de Investigaciones Científicas y Técnicas (CONICET), Fagnano 244, CP 8400 Bariloche, Río Negro Argentina; 2 Instituto de Biología Subtropical (IBS), Universidad Nacional de Misiones (UNaM), Consejo Nacional de Investigaciones Científicas y Técnicas (CONICET), Puerto Iguazú, Argentina; 3 Asociación Civil Centro de Investigaciones del Bosque Atlántico (CeIBA), Puerto Iguazú, Argentina; 4 Department of Field Conservation and Science, Bristol Zoological Society, Bristol, UK; 5 Laboratorio de Ecología de Enfermedades, Instituto de Ciencias Veterinarias del Litoral (Universidad Nacional del Litoral - Consejo Nacional de Investigaciones Científicas y Técnicas), Santa Fe, Argentina

**Keywords:** Behavioural ecology, Ecological epidemiology

## Abstract

In disease dynamics, host behaviour can both determine the quantity of parasites a host is exposed to, and be a consequence of infection. Observational and experimental studies in non-human primates have consistently found that parasitic infections result in less movement and reduced foraging, which was interpreted as an adaptive response of the host to counter infection. Variation in host nutritional condition may add complexity to the infection-behaviour relationship, and its influence may shed light on its significance. To experimentally evaluate how host activity and social relationships are affected by the interaction of parasitism and nutrition, during two years we manipulated food availability by provisioning bananas, and helminth infections by applying antiparasitic drugs, in two groups of wild black capuchin monkeys (*Sapajus nigritus*) in Iguazú National Park, Argentina. We collected faecal samples to determine the intensity of helminthic infections, as well as data on behaviour and social proximity. Individuals with unmanipulated helminth burdens foraged less than dewormed individuals only when food provisioning was low. Resting time was increased when capuchins were highly provisioned, but it did not vary according to the antiparasitic treatment. Proximity associations to other group members were not affected by the antiparasitic treatment. This is the first experimental evidence of a modulating effect of food availability on the influence of helminth infection on activity in wild primates. The findings are more consistent with an impact on host behaviour due to the debilitating effect caused by parasites than with an adaptive response to help fight infections.

## Introduction

Host behaviour is a driver of disease dynamics as it is inextricably linked to the quantity of parasites to which hosts become exposed, and may also be a consequence of infection, which in turn potentially influences disease transmission^1^. The progress of a parasitic infection depends on both exposure and the ability of the parasite to overcome the host's defences. The exposure depends on whether hosts and parasites co-occur in space and time. Hosts might display behavioural mechanisms (e.g. alteration in ranging, defecation, sleeping, dietary and social patterns) that limit encounters with parasites and thus prevent infections, the so-called avoidance strategies^2,3^. The risk of infection, therefore, depends on host behavioural traits that may reduce or increase exposure to infectious stages of a parasite^4^. High levels of exploratory behaviours, such as moving, and frequent social contact and proximity are associated with high risk of infection by parasites transmitted through direct contact or environmental contamination^5,6^.

Once a susceptible host is exposed to a parasite, both the probability of becoming infected and the eventual infection intensity depend on numerous factors, and therefore may generate a wide range of possible outcomes. By definition, parasites are detrimental to their hosts, either through direct pathological effects (e.g. tissue damage, blood loss, death)^7^, or indirectly by using the host's resources and eliciting costly immune responses, thus affecting its physiological condition^8,9^. The effect of parasites on the host's energetic balance may vary from significant^10^ to negligible^11^. Macroparasites, such as helminths, most typically produce sub-clinical effects, and milder but long-lasting impact on host fitness compared to microparasites^7^. One sub-lethal effect associated with changes in energy and nutrient budget is the infection-driven modification in host behaviour^12^. Behavioural changes associated with infection include lethargy and reduction in the time spent foraging, moving or engaging in social activities, with a corresponding increase in the time allocated to resting^13–15^. For example, wild house mice (*Mus musculus domesticus*) reduce their movement after being experimentally immune-challenged, and as a result become disconnected from their social group^16^, and high intensities of larval lungworms are associated with a decrease in the time spent foraging in bighorn rams (*Ovis canadensis*)^17^. Even though alterations in behavioural patterns have been considered merely a pathological effect exerted by the parasite, at least in part sickness behaviours can be rather interpreted as an adaptive response that allows the host to redirect energy away from disadvantageous behaviours while controlling the infection^18–20^. Less movement, reduced foraging and fewer social interactions result in less exposure to parasites/pathogens^5,6^, and avoiding new infections would be a good strategy when fighting a parasite.

Host behaviour is also linked to nutrition: it may influence the nutritional condition of the individual through changes in foraging activity^21^. At the same time, behaviour could be influenced by changes in nutrition, which is evident when food availability constrains active behaviours, such as traveling and socialising^22^ or, in the opposite direction, food enhancement leads to an increase in resting and socialising^23^. Given that the interactions between infection, nutrition and behaviour may be complex and multidirectional, understanding such interactions is challenging.

Observational studies have found some associations between parasitic infections and activity patterns in primates. For instance, wild red colobus monkeys (*Procolobus rufomitratus*) infected by whipworms (*Trichuris* sp.) in Uganda showed higher resting time and simultaneously reduced energetically demanding behaviours^24^. Similarly, in vervet monkeys (*Chlorocebus pygerythrus*) in a semi-arid region of South Africa, higher intensities of parasitic infections were linked to an increase in the time spent resting^25^. In addition, experimental studies in which monkeys were dewormed using antiparasitic drugs, have found further evidence of the impact of parasites on activity budgets (i.e. increase in resting, and decrease in foraging and moving time) and social cohesion (decreased inter-individual proximity)^6,26,27^. These consistent findings of parasite-induced activity suppression have been interpreted as evidence supporting sickness behaviour as an adaptive response that helps animals control the helminth infection. However, it may not be ruled out that the debilitating effect of parasites may be contributing to the reduction in locomotion and foraging activities. Exploring the potential effect of food availability on the infection-behaviour relationship may shed light on the significance of the observed effect. According to the “energy limitation hypothesis” ^28^, the modulation of sickness behaviour is affected by energy reserves and dependent upon a critical energy threshold. Only individuals in good nutritional conditions, with larger fat reserves, can afford sickness behaviours, such as anorexia, and loose body mass to a minimum level. Nevertheless, if energy reserves are low, the marginal benefit accrued from exhibiting sickness behavior precipitously declines, as any further decreases in body mass below a critical set point may lead to a reduced chance for recovery^28,29^. Thus, if the alterations in behaviour are adaptive, they should be less strong in malnourished individuals, as further reduction in food intake may undermine the chances of controlling the infection and recovering.

Here we contribute with results from an experimental study in which the intensity of parasitic infections and food availability were manipulated in two wild groups of black capuchin monkeys (*Sapajus nigritus*) in Iguazú National Park, Argentina. Our main goal was to investigate whether parasite removal triggers a change in activity budgets and social cohesion in capuchin monkeys, and if this effect depends on food availability.

## Materials and methods

### Study site and population

This study was conducted in Iguazú National Park, Argentina (25°40′ S, 54°30′ W), a site with a humid semi-deciduous subtropical forest and a climate characterised by a marked seasonality in day length and temperature, but not in rainfall^30^. Winter (May–August) represents the lean season, when the availability of fleshy fruits and arthropods, the bulk of the diet of black capuchins, drastically decreases at the site, while between October and January these items become abundant^21^. Specifically, with pulpy fruit productivity dropping from 1000 to 1400 g in the austral summer to 50–200 g (dry weight/ha/day) in the austral winter, black capuchins may face food shortage and depend mainly on dispersed fallback food in the latter season^31^.

At the site, black capuchin monkeys live in stable multimale-multifemale groups of 12.41 ± 7.0 (mean ± SD; n = 7) ranging from 6 to 30^32^ but occasionally reaching up to 44 individuals, with an alpha male and philopatric females that establish a linear dominance hierarchy^33^. In Iguazú, since 1991, every winter, one or two groups are provisioned with bananas on platforms for research purposes^33^. Bananas are a nutritious and highly digestible food^34^ and have become a most preferred item for black capuchins at the study site, as indicated by the high rate of food calls given by individuals when approaching the provisioning platforms^31^. In these capuchin groups, during provisioning on platforms, individual connections (network centrality) with other group members increase, but without any direct effect on parasitic infections (i.e. alterations of within-group spatial networks due to provisioning may have a limited influence in determining the characteristics of parasite infections in these monkeys)^35^.

Here we collected data from adult/subadult individuals of two groups: Macuco (15–19 adult/subadults out of 23–27 individuals) and Spot (8–12 adult/subadults out of 17–21 individuals) (Supplementary Tables S1–S2). We considered females as adults when older than five years, and subadults when four to five years old, which corresponds to the average age of female first oestrus with conception^33^. We classified males as adults when older than six years, which commonly coincides with their emigration from the natal group, and subadult when five to six years old. For most individuals, we could estimate age with a precision of one to 30 days, thanks to long-term records of all births, migrations and deaths in the study groups kept by researchers at the site.

### Experimental protocol

We manipulated food availability and the intensity of helminthic infections by provisioning with bananas, and by supplying antiparasitic drugs, respectively. Both groups were provisioned during winter (i.e. the season of food shortage for capuchins in Iguazú), but with different regimes: a high provisioning (3 bananas/platform × 3 platforms/site × 3 sites = 27 bananas), and a low provisioning regime (2–3 bananas/platform × 1 platform/site × 1–3 sites = 2–9 bananas). The low provisioning functioned as a control, reducing the chances of potential differences attributable to banana consumption/provisioning itself. To reduce the intensity of helminthic infections, we supplied approximately a randomly selected half of adults/subadults each year within each group with antiparasitic drugs, using a cocktail of ivermectin, which reduces infections by nematodes and ectoparasites, and praziquantel, which removes cestodes. Details of both experimental treatments, provisioning and parasite removal, are described in our previous study^36^ and Supplementary Tables S1–S2.

### Experimental design

We followed a split-plot experimental design consisting of four treatment groups: (1) high food provisioning and antiparasitic treatment (F+ A+), (2) high provisioning with no antiparasitic treatment (F+ A−), (3) low provisioning and antiparasitic treatment (F− A+), and (4) low provisioning with no antiparasitic treatment (F− A−). During winter 2013 we supplied the Spot group with high provisioning, and the Macuco group with a low provisioning regime. Then, in winter 2014, we switched the two provisioning regimes between the two groups: Spot received a low provisioning, while Macuco relied on a high provisioning. In winter 2013, we administered antiparasitic drugs to three individuals in Spot, and seven individuals in Macuco. In winter 2014, we supplied the treatment to four individuals in Spot, and five individuals in the Macuco (Supplementary Table S2). The random selection of the individuals to be treated with antiparasitic drugs each year was stratified by sex and social rank. Individual dominance status (high vs. low ranking; Supplementary Table S1) was assigned on the basis of a hierarchy generated by entering all observed agonistic interactions including aggressions, spatial displacements and submissive behaviours into a dominance matrix using Matman 1.1 (Noldus Information Technology 2003)^37^. Given that this was a cross-over experimental design, i.e. the treatment groups were swapped for the second year, there could have been a residual effect of antiparasitic drugs on immune function and thus individual health status and behaviour from one year to the other. However, this potential bias is unlikely since the treatment consisted of only one dose of anthelmintic drugs, monkeys were never dewormed at the study site before, and a whole year passed between the two study periods.

Parasitological and behavioural data sets (see below) were separated into three periods: (1) time prior to treatment (pre-treatment phase), from early May to early-mid July, when we collected baseline data, (2) a 7-day gap following drug administration, during which we did not consider data on parasitic infections or activity due to possible alteration of individual behaviour due to the antiparasitic drugs administration process, (3) a post-treatment phase, from mid July to late August, during which we expected changes due to the deworming in treated individuals. The duration of each phase varied among individuals since dates of drug administration varied among treated individuals within the same year and across the two study years (Supplementary Table S3). Finally, for data analyses, we classified individuals as “dewormed” (A+) only after drugs administration during the post-treatment phase, while we considered individuals as “untreated” (A−) prior to receiving treatment or if they were not treated at all during the study period^36^.

All research reported in this article complied with the protocols approved by the Ethics Committee of the Argentine Society for the Study of Mammals and adhered to the legal requirements of Argentina. All research protocols were reviewed and approved by the National Parks Administration of Argentina.

### Behavioural observations and faecal samples

During two consecutive winters (from early May to late August 2013 and 2014), we collected data on the behaviour and parasites in faeces of adult/subadult individuals in Macuco and Spot groups (Supplementary Tables S2). To calculate individual activity budgets, from dawn to dusk (approx. 12 hs/day) we collected behavioural data using focal animal sampling^38^. During 10-min focal samples, we used instantaneous recording at 1-min intervals. At each instantaneous point, we recorded the activity of the focal individual: foraging (i.e. bringing to mouth, biting, or chewing ready-to-eat foods such as fruits and plant parts, actively searching, processing or consuming foods of animal origin or certain vegetative plant parts, such as bamboo shoots and leaf bases of bromeliads), resting, moving, and social activities (including grooming, aggressions, playing and approaching), other (miscellaneous behaviours that are not included in the previous categories). In addition, at each instantaneous sample we recorded the identities of the individuals in 3 m proximity from the focal animal. We attempted to sample each focal subject within the group before beginning a new round of observations and used a criterion of spacing consecutive focal samples with at least a 1 h interval.

In order to establish the intensity of helminth infection of individuals and be able to determine the efficacy of antiparasitic treatment, we collected faecal samples opportunistically from identified adult and subadult capuchins of both groups obtaining a total of 231 faecal samples from 24 individuals during winter 2013 (mean ± SD samples per individual: 9.62 ± 3.82), and 456 faecal samples from 25 individuals during winter 2014 (18.24 ± 10.12)^36^ (Supplementary Table S1). A portion of approximately 5 g of each faecal sample was stored in a solution of 10% formalin until coprological analysis was conducted. We analysed faecal samples using a semi-quantitative flotation method with a saturated sugar solution (i.e. the Wisconsin sugar flotation method)^39^, which has proven valid for the detection of most gastrointestinal parasites’ diagnostic stages^40^. After weighing 3 g of faecal matter, we homogenised it, centrifuged and mounted the supernatant on a slide. To identify parasite structures, we used a Carl Zeiss Primo Star Microscope and took pictures with Carl Zeiss AxioCam Cc1 using the 40 × magnifier. Eggs and larvae were counted and identified on the basis of colour, shape, content and size^36^.

### Data analysis

#### Verification of antiparasitic treatment and provisioning effectiveness

We already demonstrated the efficacy of our parasite removal protocol in a previous study ^36^. In our study groups, the most prevalent parasites identified from 687 fecal samples collected from a total of 30 adult and subadult capuchins were *Filariopsis* sp. (prevalences of 54 and 72% in 2013 and 2014, respectively) and an undetermined Hymenolepididae (58–64%), followed by *Strongyloides* sp. (20–48%). The antiparasitic treatment proved to reduce the infection of the two most common parasites in our capuchin groups: the *Filariopsis* sp. and the Hymenolepididae, and reduced parasite richness by 94%^36^.

In addition, based on 46 repeated measures of body mass from 11 adult individuals (7 from Macuco group and 4 from Spot) in winter 2013, and 45 measures from 13 adults (8 from Macuco and 5 from Spot) in winter 2014, we found evidence that provisioning significantly affected individual body mass: higher provisioning with bananas resulted in an 8% increase in body mass^36^.

#### Behavioural metrics

To evaluate the effect of parasitism and nutrition on behaviour, we used two types of behavioural metrics: individual activity and social proximity. Individual activity budgets were computed from focal sample data by calculating for each individual the proportion of instantaneous points devoted to foraging, moving and resting on a daily basis. As for social proximity, we used social networks as a framework, where individuals are represented by nodes, and parasite transmission pathways can be represented by edges (i.e. connections between nodes)^41^. We computed node-based centrality measures from data on inter-individual spatial proximity collected at each instantaneous point during focal samples. This dataset was used to construct undirected association networks. We extracted node centrality indices to obtain a quantitative measure of individual centrality within a group network. For each individual, we calculated two commonly applied metrics of node centrality: “degree” = the number of links of a node) and “eigenvector centrality” = a composite value that accounts for the degree and intensity of connections that an individual has both directly and indirectly with other individuals in the network^41,42^. These centrality metrics, by quantifying the connectivity of individuals in a network, can be relevant to parasite transmission^41^. Node centrality indices were calculated using the R package *ANTs* ^43^.

#### Effect of the interaction between antiparasitic treatment and provisioning on behavior

To assess the potential interaction between antiparasitic treatment and provisioning regime on individual proportion of time devoted to foraging and resting, we used *n/k* binomial Generalised Linear Mixed Models (GLMMs) (one for each activity), where *n* = number of instantaneous records for a certain activity, and *k* = number of records belonging to all the other activity categories, for each individual on a daily basis. *Provisioning regime*, *antiparasitic treatment* and their interaction were the main predictors, while individual’s *sex*, *social rank*, and *year* were included as control variables. Individual ID and observation ID were used as random factors in all models. We used observation ID as an observation-level random effect (i.e. each data point received a unique level of a random effect) as a means to control overdispersion in our binomial proportion data^42^.

Since centrality measures are derived from a social network where all individuals are linked, they are not independent. The main method currently used to overcome this non-independency issue is the comparison of statistical models based on observed data with a distribution of null models based on randomised data^44,45^. Thus, to assess the effect of antiparasitic treatment, and its potential interaction with the provisioning regime, as well as individual factors, on node centrality measures, we constructed GLMMs on permuted association matrices using the package *ANTs*^43^. For each year, networks for each group, during pre- and post-treatment phase, were built through node-label permutations, resulting in a list of 8 matrices, each with 2000 permutations, used to calculate the centrality measures of interest (degree and eigenvector centrality) and to run permuted GLMMs. The fixed and random structures of the permuted GLMMs were selected using non-permuted GLMMs: each GLMM included as the dependent variable either degree or eigenvector centrality (modelled with a gaussian distribution) and, as fixed effects, *provisioning regime*, *antiparasitic treatment* and their interaction as the main predictors, and individual’s *sex* and *social rank*, included as control variables. The models also included individual identity (ID) as random effect.

For all GLMMs, we used Akaike information criterion (AIC) to select the best competitive models, ranking models according to AIC model weights^46^. For models using binomial distributions, we previously verified that overdispersion was not an issue ﻿(dispersion parameter ϕ < 1.2). For all models, we checked model assumptions (normality and homogeneity of residuals) and no significant violations or influential cases were detected. For models involving activities and including more than one control factor, and whenever more than one model had substantial support (i.e. when the first ranked model has a weight < 0.80), we used multimodel inference with model averaging to extract weighted parameter estimates, standard errors and 95% confidence intervals for all predictor variables within the set of candidate models. Parameter estimates and standard errors were averaged only across models in which the variable of interest appeared (conditional averaging)^46^. We performed all analyses with R software (v. 3.3.0; R Core Team 2016). In particular, we run GLMMs using R package *lme4*^47^. Model selection and model averaging were performed using the R package *MuMIn*^48^.

## Results

### Effect of antiparasitic treatment and provisioning on activity budgets

**Figure 1:**
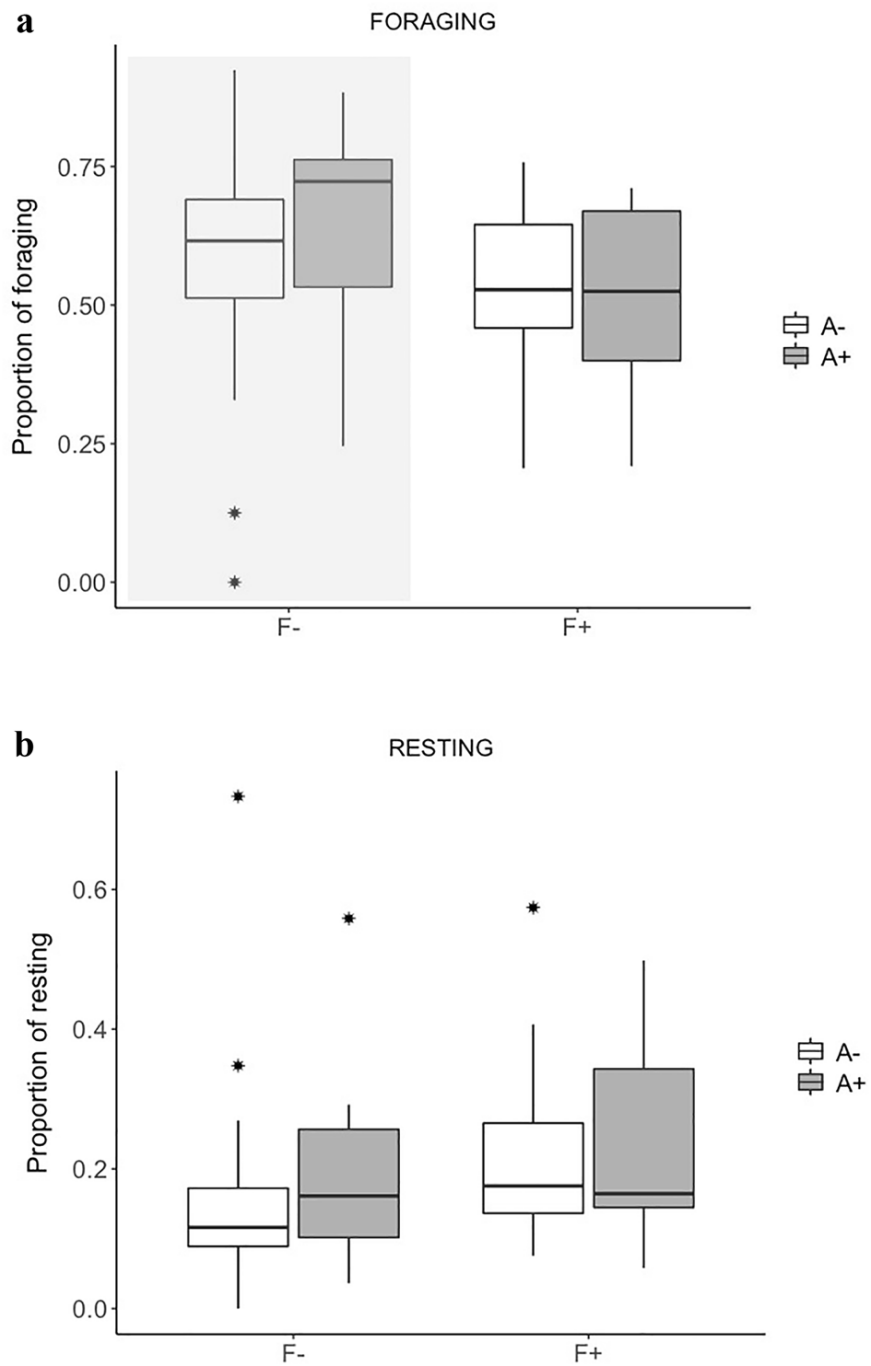
Boxplots illustrating individual black capuchin monkeys’ mean daily budgets for foraging and resting according to the antiparasitic treatment and provisioning regime. F+: High Provisioning, F−: Low Provisioning, A+: Dewormed individuals, A−: Untreated individuals. Bottom and top of the box show the 25th and 75th percentiles and whiskers show the largest data point that is less than 1.5 times the interquartile range above the 75th percentile. Asterisks indicate outliers. Grey = treated (A+), white = untreated individuals (A−). Significant differences (CI excluding 0) are shaded.

We calculated activity budgets on the basis of 1579 daily data points (16,898 instantaneous point samples) of 22 individuals (mean number of daily points per individual ± SD = 71.77 ± 30.44) collected in 2013 (pre-treatment phase: 7.90 ± 5.09; post-treatment phase: 32.82 ± 13.30) and 2014 (pre-treatment: 18.89 ± 8.99; post-treatment: 17.47 ± 8.13) (Supplementary Tables S1–S2). We found an interaction between provisioning and antiparasitic treatment’s effects on foraging, i.e. dewormed capuchins spent proportionally more time foraging (75% higher) than untreated ones when provisioning was low (odds ratio (OR) = 0.57). There was no difference in the overall proportion of time resting between dewormed and untreated individuals, but resting was increased in all capuchins when provisioning was high (OR = 1.28) (Fig. 1, Table 1, Supplementary Table S4).

**Table 1:** Results of Generalised Linear Mixed Models (GLMMs) including all fixed factors for each of two response variables, foraging and resting activities.

Response variable	Explanatory variables	Parameter estimate ± SE	95% confidence intervals	
			Lower	Upper
Foraging	Intercept	1.121 ± 0.169	0.790	1.451
	Provis (F+)	− 0.187 ± 0.118	− 0.418	0.043
	Antipar (A+)	0.239 ± 0.153	− 0.061	0.538
	**Year (2014)**	**− 1.152 ± 0.092**	**− 1.334**	**− 0.972**
	**Rank (DOM)**	**− 0.431 ± 0.157**	**− 0.738**	**− 0.124**
	**Sex (F)**	**0.353 ± 0.155**	**0.048**	**0.657**
	**Provis(F+):Antipar(A+)**	**− 0.569 ± 0.225**	**− 1.011**	**− 0.128**
Resting	Intercept	− 2.585 ± 0.165	− 2.908	− 2.261
	**Provis (F+)**	**0.248 ± 0.132**	**0.159**	**0.639**
	Antipar (A+)	0.316 ± 0.079	− 0.012	0.508
	**Year (2014)**	**0.518 ± 0.112**	**0.298**	**0.739**
	**Rank (DOM)**	**0.328 ± 0.121**	**0.089**	**0.566**
	**Sex (F)**	**− 0.370 ± 0.128**	**− 0.622**	**− 0.118**
	Prov (F+):Antipar (A+)	− 0.010 ± 0.240	− 0.482	0.461

Levels of categorical predictors included in the intercept are the following: Low Provisioning (F −), Antiparasitic Untreated (A −), Year (2014), Sex (M), and social Rank (SUB). The reference levels not included in the intercept are High Provisioning (F+), Antiparasitic Dewormed (A+), Year (2013), Sex (F) and social Rank (DOM). Estimates (± SE) and 95% confidence interval for the parameters of explanatory variables describing variation in individual activity budgets are provided. See methods for details. Confidence intervals excluding zero are in reported in bold.

**Figure 2:**
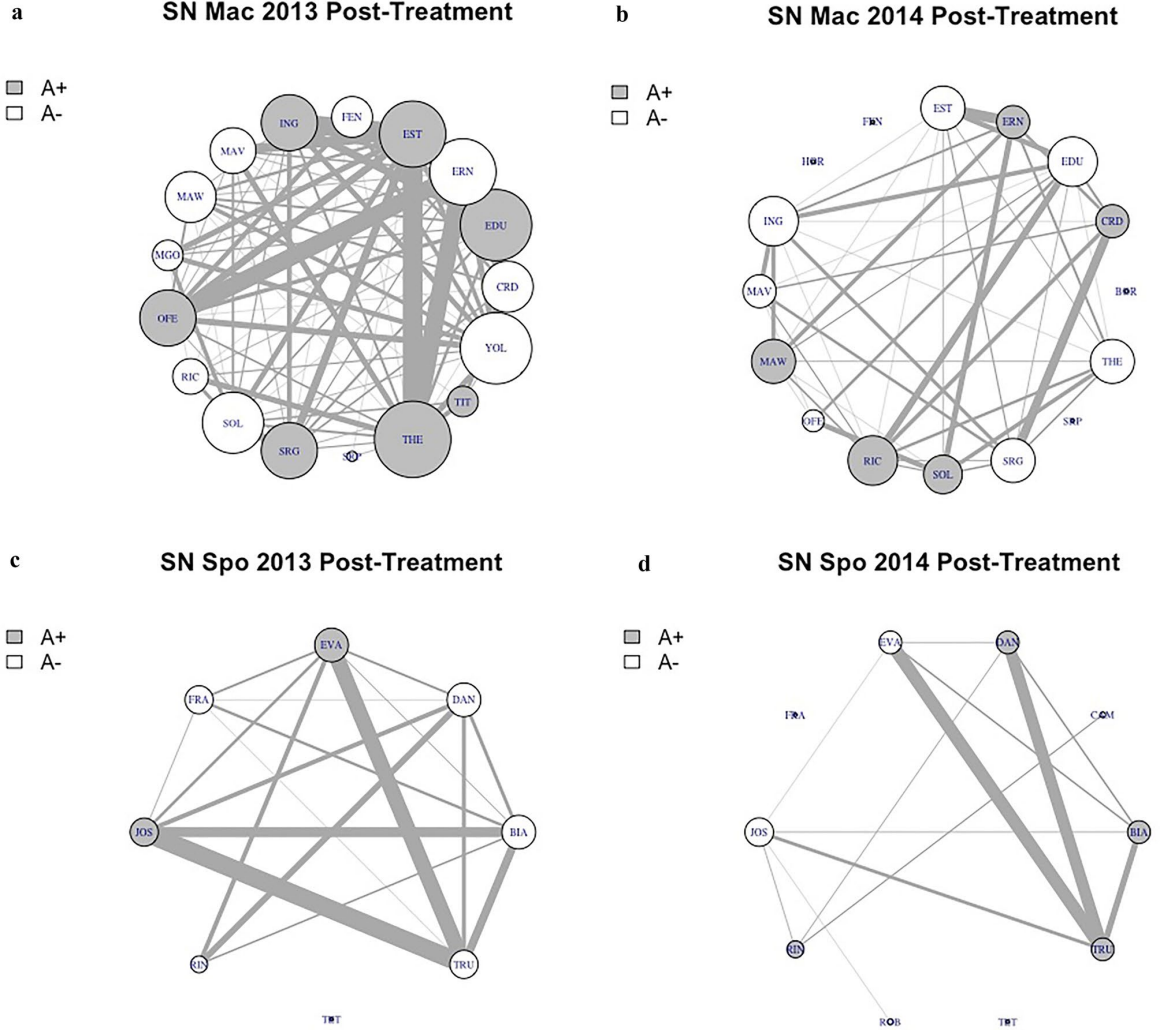
Post-treatment social (proximity) networks (SN) for (**a, b**) Macuco and (**c, d**) Spot groups in 2013 and 2014. Nodes represent individuals and edges (connections between nodes) represent the proximity relationships. Node sizes represent the variation in the number of associations (degree), the larger the higher, while line thickness represents the strength of associations between two nodes, the thicker the strongest. Grey = treated (A+), white = untreated individuals (A−).

Proximity networks and centrality indices for individuals were constructed based on 1567 dyadic associations involving adult and subadult group members. In particular, for Macuco group, we counted on 718 associations in 2013 (89 in the pre-treatment and 629 in the post-treatment phase) and 491 in 2014 (265 pre- and 226 post-treatment). For Spot, we relied on a set of 220 associations in 2013 (41 pre- and 179 post-treatment), and 138 in 2014 (49 pre- and 89 post-treatment). Regardless of the provisioning regime, dewormed capuchin individuals tended to have more and more important associations with other adults or subadults compared to untreated individuals (Fig. 2), however the observed effect of treatment on the degree or the eigenvector centrality was not more pronounced than the same effect from a set of null models derived from randomised network data (Table 2, Supplementary Table S5).

**Table 2:** Results of permuted GLMMs performed to explain the variance of social centrality.

Response variable	Explanatory variables	Parameter estimate ± SE	95% Confidence Intervals	
			Lower	Upper
Degree	Intercept	2.429 ± 0.899	3.917	5.453
	Provis (F+)	1.026 ± 0.667	0.304	1.135
	Antipar (A+)	4.594 ± 1.047	2.262	5.159
	**Rank (DOM)**	**1.061 ± 1.071**	**− 2.156**	**− 0.204**
	**Sex (F)**	**1.810 ± 0.982**	**− 1.306**	**0.653**
	Provis(F+):Antipar(A+)	− 4.184 ± 1.663	− 5.771	− 1.260
Eigenvector	Intercept	0.044 ± 0.079	0.212	0.386
	Provis (F+)	0.103 ± 0.040	0.095	0.107
	Antipar (A+)	0.176 ± 0.051	− 0.050	0.196
	**Rank (DOM)**	**0.282 ± 0.100**	**− 0.113**	**0.122**
	**Sex (F)**	**0.235 ± 0.088**	**− 0.120**	**0.114**

All permuted GLMMs included all fixed factors for each of two variables: degree and eigenvector centrality. Levels of categorical predictors included in the intercept are the following: Low Provisioning (F −), Antiparasitic Untreated (A −), Year (2014), Sex (M), and social Rank (SUB). The reference levels not included in the intercept are High Provisioning (F+), Antiparasitic Dewormed (A+), Year (2013), Sex (F) and social Rank (DOM). Estimates (± SE) and 95% confidence interval for the parameters of explanatory variables describing variation in individual centrality are provided. See methods for details. Confidence intervals excluding the estimate are in reported in bold.

## Discussion

This study represents the first experimental test of the effect of the interaction between parasitic infection and food availability on the behaviour of a wild primate species. We found an interaction between food provisioning and antiparasitic treatment in their effects on foraging: capuchins with natural infection intensities spent less time foraging than dewormed ones when provisioning was low, suggesting that the modification of the behavioural pattern caused by parasites is likely a consequence of the debilitating effect caused by the combination of parasites and poor nutrition, rather than an adaptive response to parasitism. Dewormed individuals did not show higher degree of spatial proximity to other group members, compared to untreated ones, indicating a negligible impact of parasites on capuchins’ social behaviour.

The behavioural manifestations of sickness are considered an adaptive response to aid the host in clearance of the infectious agent^49,50^. Cytokines involved in the immune response act on the brain to generate fever, anorexia, and reduction in social, exploratory, and sexual behaviours^49,50^. Nonetheless, being sick has a significant energetic cost. The induction of anorexia in an organism that has a debilitating disease is maladaptive when the host is in poor body condition. In fact, past research showed that body fat stores and leptin levels (a hormone that regulates the energy balance) influence the magnitude of immune responses and sickness behaviours^49^. For instance, rats that were fasted and injected with a dose of LPS (an antigen from the cell wall of gram-negative bacteria that induces immune response and sickness behaviour) showed an attenuated fever response as compared to rats that are fed *ad libitum*^51^. In addition, mice provided with a calorie-restricted diet showed no anorexia when treated with LPS^52^. Also, rats that were fasted for 48 hs showed a 30% reduction in hypothalamic cytokines^53^, and rats displaying diet-induced obesity showed greater levels of circulating hypothalamic cytokines compared to lean controls after LPS injection^54^, suggesting that changes in energy stores may alter cytokine production. For all the above, we predicted that the impact of the antiparasitic drugs on foraging behaviour would be greater in monkeys that received high levels of supplementation with bananas, but we observed the opposite.

It is noteworthy that the immune response generated by helminths (Th2) is very different and is mediated by different cytokines than the one generated by bacteria and viruses (Th1)^55^. The literature that explores the adaptive value of sickness behaviour largely focuses on infections with microparasites, which cause fever and the accompanying behavioural alterations described above. Observational and experimental studies carried out in wild primates investigated the infection-behaviour interaction interpreting the effect of infection with helminths as sickness behaviour. Red colobus (*Procolobus rufomitratus*) have been found to increase resting, decrease energetically costly activities, and switched behaviours less frequently when whipworm positive^24^. Vervet monkeys (*Chlorocebus aethiops*), following treatment with ivermectin, decreased the amount of time devoted to resting by 75.8% while increasing the time traveling by 53.4%^27^. Other two experimental studies on semi-free ranging primate populations of red-capped mangabeys (*Cercocebus torquatus*) and Barbary macaques (*Macaca sylvanus*) had similar results: following the antiparasitic treatment, individuals’ stress hormones levels (cortisol or glucocorticoid metabolites) and frequency of active behaviours increased^6,26^. A more recent study investigating the behavioural response of other vervet monkeys (*Chlorocebus pygerytrhus*) to the infection with gastrointestinal parasites has also taken into account food availability, using the Normalised Difference Vegetation Index—NDVI—in groups’ home ranges as a proxy, although not testing the potential interaction between food availability and parasitism. While increased resting time and decreased feeding time was associated to *Protospirura* sp., an opposite pattern, i.e. a decrease in resting time, was found for *Trychostrongylus* sp., showing that behavioural changes may be parasite-specific and depend on co-infection. Food availability was the most influencing factor affecting vervets’ behaviour in this semi-arid region of South Africa, with high food availability predicting an increase in the probability of resting and a decrease in the probability of feeding^25^. All these studies have formulated hypotheses and predictions, and interpreted results following the premise that parasite-induced changes in behaviour are the result of an adaptive response that allows the host to conserve energy and fight infections^19^.

Here we experimentally evaluated the effect of anthelmintic drugs on activity patterns at two manipulated levels of food availability, finding that the deworming had an effect but only in the group with low provision of bananas, which runs counter to what would be expected of an adaptive sickness behaviour. The fact that less nourished individuals with natural helminth infections foraged less than dewormed ones suggests that the debilitating impact of parasites was stronger in those that were weaker.

So far, studies using anthelmintic treatments in a variety of species have revealed a great variation and complexity of the impact exerted by helminths, providing relatively strong evidence of parasite-induced reduction in foraging in domestic animals^14,56^, but much ambiguous results in wildlife studies^57^. For example, evidence of parasite-induced reduction in foraging time was observed in Grant’s gazelles (*Nanger granti*)^58^ infected with strongyle nematodes and lungworms, whereas it was not found in Soay sheep (*Ovis aries*)^59^ and grey kangaroos (*Macropus giganteus*) that harbour strongyle nematodes^60^, and was in the opposite direction (i.e. parasites inducing higher food consumption) in sticklebacks (*Gasterosteus aculeatus*) that were parasitised by the cestode *Schistocephalus solidus*^61^. Similarly, in Cuban tree frogs (*Osteopilus septentrionalis*) on a high resource diet, individuals parasitised by skin-penetrating gut nematode *Aplectana* sp. consumed more food than non-parasitised frogs^62^.

Anorexia, as a response to gastrointestinal parasitic infection, has been seen as paradoxical, as parasites frequently impose an increased energetic demand on hosts, leading to loss of body condition, and decreased growth rate and reproductive success^56^. However, some argument for the potential adaptive significance of anorexia is that, together with lethargy, it may act to deviate resources to trigger an immune response by the host, while allowing, at the same time, the host to avoid further ingestion of infectious stages of parasites^14,19^. As immune responses can be energetically costly to the host, they depend on the resources available^63^.

Indeed, in previous work, we demonstrated that when capuchin monkeys are well nourished, with strong and functional immune defences, they are more resistant to parasitic infections compared with less nourished individuals^36^. However, we could not demonstrate any effect of the antiparasitic treatment, or its interaction with food availability, on host physical condition. The lack of these effects may have suggested that these monkeys are tolerant to the existing intensities of parasite infections since individuals maintain their body mass despite increased parasite infections. However, we acknowledged we could not rule out that parasites might affect other aspects, such as host activity budgets and social relationships^26^, which in turn could affect reproduction and fitness^27^. The results obtained in this study showed that this was the case, and also that the reduction in foraging caused by parasites was greater the lower the food availability, which suggests that those individuals may face difficulties in maintaining their body condition. Likely, in times of severe food shortage, infected capuchins may even begin to suffer from weight loss. An alternative explanation of the modulating effect of food availability may be that, as highly supplemented capuchins are more resistant to parasites^36^, they never reach threshold infection levels that trigger a significant change in their energy balance and behaviour.

Although we found support to parasite-induced reduction in foraging, we did not find any effect of antiparasitic treatment on resting budgets in capuchins. Individuals rested more when highly provisioned, probably as a result of the increased consumption of high-energy resources^23^, but regardless of the individual infection status. Other studies investigating parasite-induced behavioural changes also found that parasitism can impact some behavioural traits but not others^24,27^ and it has been acknowledged that alterations in behaviour may show flexibility in their expression^63^.

Despite the evidence of a difference in activity associated with antiparasitic treatment, untreated individuals were not avoided by conspecifics compared to dewormed ones. Avoidance of infected partners, as a behavioural parasite avoidance strategy, has been documented in a wide array of species, from lobsters^64^ to mandrills^65^, indicating that different animals can detect infected conspecifics, mainly via chemical or olfactory cues^4^. In our study, although black capuchins decrease foraging when naturally infected, they may not show other apparent signs of disease, which is likely the reason why infected individuals may go undetected and are not actively avoided by conspecifics.

One of the main flaws of this study is the small sample size of dewormed individuals, which could have affected the power of some statistical analyses. In addition, the unknown variation in the potential costs associated to infections with the identified parasites may have influenced our results in a way that we cannot predict: likely, low pathogenic parasites are well tolerated by capuchins and probably do not trigger conspicuous behavioural changes in hosts, while highly pathogenic ones may have deleterious effects. Future studies should address the role of different parasites in shaping capuchins’ behaviour, shedding further light into the complexity of parasites-host behaviour relationship.

In conclusion, our study is the first to experimentally demonstrate that food resources affect the relationship between parasites and individual behaviour in a wild primate species. Together with our previous work, these results highlight the importance of nutrition in modulating the response of hosts to parasites. Since the availability of food resources, as well as exposure to parasites, vary from year to year, the impact of parasites may see profound temporal changes and might need long term data to be appropriately assessed.

## Supplementary Information

Supplementary Information 1.

Supplementary Information 2.

## Data Availability

The authors declare that all data supporting the findings of this study are available within the paper and its Supplementary Information files.

## References

[1] Moore J (2012). An overview of parasite-induced behavioral alterations - and some lessons from bats. J. Exp. Biol..

[2] Nunn CL, Altizer S (2006). Infectious Diseases in Primates: Behavior, Ecology and Evolution.

[3] Hutchings MR, Athanasiadou S, Kyriazakis I, Gordon IJ (2003). Nutrition and Behaviour Group Symposium on ‘Exploitation of medicinal properties of plants by animals and man through food intake and foraging behaviour’: Can animals use foraging behaviour to combat parasites?. Proc. Nutr. Soc..

[4] Hawley DM, Etienne RS, Ezenwa VO, Jolles AE (2011). Does animal behavior underlie covariation between hosts’ exposure to infectious agents and susceptibility to infection? Implications for disease dynamics. Integr. Comp. Biol..

[5] Rimbach R (2015). Brown spider monkeys (*Ateles hybridus*): a model for differentiating the role of social networks and physical contact on parasite transmission dynamics. Philos. Trans. R. Soc. B Biol. Sci..

[6] Friant S, Ziegler TE, Goldberg TL (2016). Changes in physiological stress and behaviour in semi-free-ranging red-capped mangabeys (*Cercocebus torquatus*) following antiparasitic treatment. Proc. R. Soc. B Biol. Sci..

[7] Hudson PJ, Dobson AP, Grenfell BT, Dobson AP (1995). Macroparasites: Observed patterns in naturally fluctuating animal populations. Ecology of infectious diseases in natural populations.

[8] Murray DL, Lloyd BK, Cary JR (1998). Do parasitism and nutritional status interact to affect production in snowshoe hares?. Ecology.

[9] Coop RL, Holmes PH (1996). Nutrition and parasite interaction. Int. J. Parasitol..

[10] Møller AP, de Lope F, Moreno J, González G, Pérez JJ (1994). Ectoparasites and host energetics: House martin bugs and house martin nestlings. Oecologia.

[11] Munger JC, Karasov WH (1989). Sublethal parasites and host energy budgets: Tapeworm infection in white-footed mice. Ecology.

[12] Hicks O (2018). The energetic cost of parasitism in a wild population. Proc. R. Soc. B Biol. Sci..

[13] Sánchez CA (2018). On the relationship between body condition and parasite infection in wildlife: A review and meta-analysis. Ecol. Lett..

[14] Kyriazakis I, Tolkamp BJ, Hutchings MR (1998). Towards a functional explanation for the occurrence of anorexia during parasitic infections. Anim. Behav..

[15] Hart BL (1990). Behavioral adaptations to pathogens and parasites: Five strategies. Neurosci. Biobehav. Rev..

[16] Lopes PC, Block P, König B (2016). Infection-induced behavioural changes reduce connectivity and the potential for disease spread in wild mice contact networks. Sci. Rep..

[17] Pelletier F, Festa-Bianchet M (2004). Effects of body mass, age, dominance and parasite load on foraging time of bighorn rams. Ovis canadensis. Behav. Ecol. Sociobiol..

[18] Bonneaud C (2003). Assessing the cost of mounting an immune response. Am. Nat..

[19] Hart BL (1991). The behavior of sick animals. Vet. Clin. North Am. Small Anim. Pract..

[20] Poulin R (1994). Meta-analysis of parasite-induced behavioural changes. Anim. Behav..

[21] Janson CH, Janson CH, Norconk MA, Rosenberger AL, Garber PA (1996). Toward an experiemental socioecology of primates. Examples from Argentine brown capuchin monkeys (*Cebus apella nigritus*). Adaptive Radiations of Neotropical Primates.

[22] Robinson JG (1986). Seasonal variation in use of time and space by the wedge-capped capuchin monkey, *Cebus olivaceus*: Implications for foraging theory. Smithson. Contrib. Zool..

[23] Saj T, Sicotte P, Paterson JD (1999). Influence of human food consumption on the time budget of vervets. Int. J. Primatol..

[24] Ghai RR, Fugère V, Chapman CA, Goldberg TL, Davies TJ (2015). Sickness behaviour associated with non-lethal infections in wild primates. Proc. Biol. Sci..

[25] Blersch R (2021). Sick and tired: Sickness behaviour, polyparasitism and food stress in a gregarious mammal. Behav. Ecol. Sociobiol..

[26] Müller-Klein N (2019). Physiological and social consequences of gastrointestinal nematode infection in a nonhuman primate. Behav. Ecol..

[27] Chapman CA (2016). Social behaviours and networks of vervet monkeys are influenced by gastrointestinal parasites. PLoS ONE.

[28] Owen-Ashley NT, Wingfield JC (2007). Acute phase responses of passerine birds: characterization and seasonal variation. J. Ornithol..

[29] Owen-Ashley NT, Wingfield JC (2006). Seasonal modulation of sickness behavior in free-living northwestern song sparrows (*Melospiza melodia morphna*). J. Exp. Biol..

[30] Janson CH, Di Bitetti MS (1997). Experimental analysis of food detection in capuchin monkeys: Effects of distance, travel speed, and resource size. Behav. Ecol. Sociobiol..

[31] Di Bitetti, M. S. Food-associated calls in the tufted capuchin monkey (*Cebus apella*). PhD Thesis. (Stony Brook University, New York, 2001).

[32] Di Bitetti MS, Janson CH (2001). Reproductive socioecology of tufted capuchins (*Cebus apella nigritus*) in Norteastern Argentina. Int. J. Primatol..

[33] Janson C, Baldovino MC, Di Bitetti M, Kappeler PM, Watts DP (2012). The group life cycle and demography of brown capuchin monkeys (*Cebus [apella] nigritus*) in Iguazú National Park, Argentina. Long-Term Field Studies of Primates.

[34] Robinson, J. C. & Galán Saúco, V. *Bananas and plantains*. (Crop production science in horticulture series N. 19, CAB International, 2010). 10.1079/9781845936587.0000

[35] Tiddi B, Pfoh R, Agostini I (2019). The impact of food provisioning on parasite infection in wild black capuchin monkeys: A network approach. Primates.

[36] Agostini I, Vanderhoeven E, Di Bitetti MS, Beldomenico PM (2017). Experimental testing of reciprocal effects of nutrition and parasitism in wild black capuchin monkeys. Sci. Rep..

[37] de Vries H, Netto WJ, Hanegraaf PLH (1993). Matman: a program for the analysis of sociometric matrices and behavioural transition matrices. Behaviour.

[38] Martin P, Bateson P (1993). Measuring Behaviour.

[39] Cox DD, Todd AC (1962). Survey of gastrointestinal parasitism in Wisconsin dairy cattle. J. Am. Vet. Med. Assoc..

[40] Ballweber LR, Beugnet F, Marchiondo AA, Payne PA (2014). American association of veterinary parasitologists’ review of veterinary fecal flotation methods and factors influencing their accuracy and use—Is there really one best technique?. Vet. Parasitol..

[41] Godfrey SS (2013). Networks and the ecology of parasite transmission: a framework for wildlife parasitology. Int. J. Parasitol. Parasites Wildl..

[42] Sosa S, Sueur C, Puga-Gonzalez I (2020). Network measures in animal social network analysis: Their strengths, limits, interpretations and uses. Methods Ecol. Evol..

[43] Sosa S (2020). A multilevel statistical toolkit to study animal social networks: The Animal Network Toolkit Software (ANTs) R package. Sci. Rep..

[44] Croft DP, Madden JR, Franks DW, James R (2011). Hypothesis testing in animal social networks. Trends Ecol. Evol..

[45] Farine DR (2013). Animal social network inference and permutations for ecologists in R using asnipe. Methods Ecol. Evol..

[46] Burnham, K. P. & Anderson, D. R. *Model Selection and Multimodel Inference: A Practical Information-Theoretic Approach (2nd ed)*. *Ecological Modelling* (Springer, 2002). 10.1016/j.ecolmodel.2003.11.004

[47] Bates D, Maechler M, Bolker B, Walker S (2015). Fitting linear mixed-effects models using lme4. J. Stat. Softw..

[48] Barton, K. MuMIn: Multi-model inference. R package version 1.15.6. 63 (2016). citeulike:11961261

[49] Carlton ED, Demas GE, French SS (2012). Leptin, a neuroendocrine mediator of immune responses, inflammation, and sickness behaviors. Horm. Behav..

[50] Tizard I (2008). Sickness behavior, its mechanisms and significance. Anim. Health Res. Rev..

[51] Inoue W, Luheshi GN (2010). Acute starvation alters lipopolysaccharide-induced fever in leptin-dependent and -independent mechanisms in rats. Am. J. Physiol. Regul. Integr. Comp. Physiol..

[52] Macdonald L, Radler M, Paolini AG, Kent S (2011). Calorie restriction attenuates LPS-induced sickness behavior and shifts hypothalamic signaling pathways to an antiinflammatory bias. Am. J. Physiol. Regul. Integr. Comp. Physiol..

[53] Wisse BE (2004). Physiological regulation of hypothalamic IL-1β gene expression by leptin and glucocorticoids: implications for energy homeostasis. Am. J. Physiol. Endocrinol. Metab..

[54] Pohl J, Woodside B, Luheshi GN (2009). Changes in hypothalamically mediated acute-phase inflammatory responses to lipopolysaccharide in diet-induced obese rats. Endocrinology.

[55] Bretscher P (2019). On analyzing how the Th1/Th2 phenotype of an immune response is determined: classical observations must not be ignored. Front. Immunol..

[56] Poppi DP, Sykes AR, Dynes RA (1990). The effect of endoparasitism on host nutrition - the implications for nutrient manipulation. Proc. New Zeal. Soc. Anim. Prod..

[57] Coulson G, Cripps JK, Garnick S, Bristow V, Beveridge I (2018). Parasite insight: assessing fitness costs, infection risks and foraging benefits relating to gastrointestinal nematodes in wild mammalian herbivores. Philos. Trans. R. Soc. B Biol. Sci..

[58] Worsley-Tonks KEL, Ezenwa VO (2015). Anthelmintic treatment affects behavioural time allocation in a free-ranging ungulate. Anim. Behav..

[59] Jones OR, Anderson RM, Pilkington JG (2006). Parasite-induced anorexia in a free-ranging mammalian herbivore: An experimental test using Soay sheep. Can. J. Zool..

[60] Cripps JK, Martin JK, Coulson G (2016). Anthelmintic treatment does not change foraging strategies of female eastern grey kangaroos, *Macropus giganteus*. PLoS ONE.

[61] Giles N (1987). Predation risk and reduced foraging activity in fish: experiments with parasitized and non-parasitized three-spined sticklebacks, *Gasterosteus aculeatus* L.. J. Fish Biol..

[62] Knutie SA, Wilkinson CL, Wu QC, Ortega CN, Rohr JR (2017). Host resistance and tolerance of parasitic gut worms depend on resource availability. Oecologia.

[63] Lopes PC, French SS, Woodhams DC, Binning SA (2021). Metabolic response of dolphins to short-term fasting reveals physiological changes that differ from the traditional fasting model. J. Exp. Biol..

[64] Behringer DC, Butler MJ, Shields JD (2006). Ecology: Avoidance of disease by social lobsters. Nature.

[65] Poirotte C (2017). Mandrills use olfaction to socially avoid parasitized conspecifics. Sci. Adv..

