# Supplementary material for: Experimental evidence of parasite-induced behavioural alterations modulated by food availability in wild capuchin monkeys
Source: Sci Rep. 2023 Feb 22;13:3083. doi: 10.1038/s41598-023-30262-2 (PMC9947137; doi:10.1038/s41598-023-30262-2)
Supplement: Supplementary file 1 — Supplementary Information 1. [file 41598_2023_30262_MOESM1_ESM.docx]

***SUPPORTING INFORMATION***

*Title*: Experimental evidence of parasite-induced behavioural alterations modulated by food availability in wild capuchin monkeys

*Authors*: Ilaria Agostini^1,2,3^, Ezequiel Vanderhoeven^2,3^, Romina Pfoh^3^, Barbara Tiddi^4^, Pablo M. Beldomenico^5^

^1^ Parque Nacional Nahuel Huapi (CENAC-APN), Consejo Nacional de Investigaciones Científicas y Técnicas (CONICET), Bariloche, Río Negro, Argentina.

^2^ Instituto de Biología Subtropical (IBS), Universidad Nacional de Misiones (UNaM), Consejo Nacional de Investigaciones Científicas y Técnicas (CONICET), Argentina.

^3^ Asociación Civil Centro de Investigaciones del Bosque Atlántico (CeIBA), Argentina.

^4^ Department of Field Conservation and Science, Bristol Zoological Society, Bristol, U.K.

^5^Laboratorio de Ecología de Enfermedades, Instituto de Ciencias Veterinarias del Litoral (Universidad Nacional del Litoral - Consejo Nacional de Investigaciones Científicas y Técnicas), Argentina.

Table S1. Summary of study groups’ composition, individuals’ age-sex class and social rank, provisioning regime (F+: high; F-: low) and antiparasitic treatment (A+: dewormed; A-: untreated) received, as well as number of faecal samples, behavioural instantaneous point samples and daily point samples (from focals) obtained in 2013 and 2014 for MACUCO and SPOT group.

| **Group/**  **Year** | **Ind. ID** | **Age-sex** | **Rank** | **Provisioning** | **Antiparasitic**  **treatment** | **# Fecal**  **samples** | **# Instant. point samples (# daily point samples)** |
| --- | --- | --- | --- | --- | --- | --- | --- |
| **MACUCO**  **2013** | CRD | ADF | Low | F- | A- | 8 | 741 (48) |
|  | EST | ADF | High | F- | A+ | 10 | 534 (49) |
|  | ING | ADF | Low | F- | A+ | 9 | 700 (54) |
|  | MAW | ADF | Low | F- | A- | 9 | 446 (43) |
|  | OFE | ADF | Low | F- | A+ | 13 | 446 (44) |
|  | SOL | ADF | Low | F- | A- | 7 | 413 (45) |
|  | THE | ADF | High | F- | A+ | 14 | 711 (49) |
|  | YOL | ADF | Low | F- | A- | 10 | 440 (49) |
|  | EDU | ADM | High | F- | A+ | 18 | 242 (24) |
|  | ERN | ADM | High | F- | A- | 14 | 349 (29) |
|  | GAB | ADM | Low | F- | A- | - | - |
|  | MGO | ADM | Low | F- | A- | 11 | 100 (15) |
|  | SRG | ADM | High | F- | A+ | 13 | 167 (19) |
|  | SRP | ADM | Low | F- | A- | 9 | - |
|  | TIT | ADM | Low | F- | A+ | 5 | 101 (13) |
|  | FEN | SUBF | Low | F- | A- | - | - |
|  | DAL | SUBM | Low | F- | A- | 2 | - |
|  | MAV | SUBM | Low | F- | A- | 3 | 121 (13) |
|  | RIC | SUBM | Low | F- | A- | 4 | 122 (15) |
|  |  |  |  |  | **TOTAL** | **159** | **5633 (509)** |
| **MACUCO**  **2014** | CRD | ADF | Low | F+ | A+ | 31 | 338 (45) |
|  | EST | ADF | High | F+ | A- | 19 | 312 (40) |
|  | FEN | ADF | Low | F+ | A- | 18 | - |
|  | ING | ADF | Low | F+ | A- | 25 | 616 (61) |
|  | MAW | ADF | Low | F+ | A+ | 21 | 752 (65) |
|  | OFE | ADF | Low | F+ | A- | 20 | 491 (44) |
|  | SOL | ADF | Low | F+ | A+ | 17 | 440 (42) |
|  | THE | ADF | High | F+ | A- | 16 | 502 (51) |
|  | EDU | ADM | High | F+ | A- | 25 | 314 (37) |
|  | ERN | ADM | High | F+ | A+ | 30 | 538 (43) |
|  | SRG | ADM | High | F+ | A- | 34 | 284 (28) |
|  | BOR | ADM | Low | F+ | A- | 10 | - |
|  | HOR | ADM | Low | F+ | A- | 8 | - |
|  | PAC | ADM | Low | F+ | A- | 2 | - |
|  | ESB | SUBF | Low | F+ | A- | - | - |
|  | MOR | SUBF | Low | F+ | A- | - | - |
|  | MAV | SUBM | Low | F+ | A- | 12 | 124 (13) |
|  | RIC | SUBM | Low | F+ | A+ | 37 | 559 (48) |
|  |  |  |  |  | **TOTAL** | **305** | **5270 (517)** |

| **Group/ Year** | **Ind. ID** | **Age-sex** | **Rank** | **Provisioning** | **Antiparasitic**  **treatment** | **# Fecal**  **samples** | **# Instant. point samples**  **(# daily point samples)** |
| --- | --- | --- | --- | --- | --- | --- | --- |
| **SPOT**  **2013** | BIA | ADF | High | F+ | A- | 10 | 967 (64) |
|  | DAN | ADF | Low | F+ | A- | 8 | 615 (55) |
|  | EVA | ADF | Low | F+ | A+ | 14 | 979 (71) |
|  | JOS | ADF | Low | F+ | A+ | 12 | 704 (62) |
|  | RIN | ADM | High | F+ | A- | 10 | 384 (37) |
|  | TET | ADM | High | F+ | A+ | 11 | 261 (35) |
|  | TRU | ADM | High | F+ | A- | 7 | 704 (55) |
|  | FRA | SUBF | Low | F+ | A- | - | - |
|  |  |  |  |  | **TOTAL** | **72** | **4614 (379)** |
| **SPOT**  **2014** | BIA | ADF | High | F- | A+ | 36 | 400 (44) |
|  | DAN | ADF | Low | F- | A+ | 9 | 182 (29) |
|  | EVA | ADF | Low | F- | A- | 19 | 273 (33) |
|  | FRA | ADF | Low | F- | A- | 1 | - |
|  | JOS | ADF | Low | F- | A- | 13 | 226 (27) |
|  | RIN | ADM | High | F- | A+ | 17 | 89 (15) |
|  | TET | ADM | High | F- | A- | 4 | 37 (6) |
|  | TRU | ADM | High | F- | A+ | 12 | 174 (20) |
|  | CAM | SUBM | Low | F- | A- | - | - |
|  | ROB | SUBM | Low | F- | A- | 20 | - |
|  |  |  |  |  | **TOTAL** | **151** | **1381 (174)** |

ADF = adult female; ADM = adult male; SUBF = subadult female; SUBM = subadult male.

Table S2. Details of group composition, experimental study design and sampling dates in 2013 and 2014.

| **Year** | **Group ID** | **2013 (winter)** | **2014 (winter)** |
| --- | --- | --- | --- |
| Group composition | Macuco | 15 AD (8 F, 7 M)  4 SUB (1 F, 3 M)  8 IMM | 11 AD (8 F, 3 M)  4 SUB (2 F, 2 M)  9 IMM |
|  | Spot | 7 AD (4 F, 3 M)  1 SUB (1 F)  9 IMM | 10 AD (5 F, 5 M)  2 SUB (2 M)  9 IMM |
| Provisioning treatment | Macuco | All individuals (Low)  66 days | All individuals (High)  60 days |
|  | Spot | All individuals (High)  80 days | All individuals (Low)  29 days |
| Antiparasitic treatment  (individuals) | Macuco | Dewormed: 4 ADF,  3 ADM  Untreated: 4 ADF,  5 ADM, 1 SUBF, 2 SUBM | Dewormed: 3 ADF, 1 ADM,  1 SUBM  Untreated: 4 ADF, 6 ADM*,  2 SUBF, 1 SUBM |
|  | Spot | Dewormed: 2 ADF,  1 ADM  Untreated: 2 ADF,  2 ADM, 1 SUBF | Dewormed: 2 ADF, 2 ADF  Untreated: 3 ADF,  3 ADM, 2 SUBM |
| Fecal sample collection | Macuco | May 6 – Aug 31 | May 13 – Aug 27 |
|  | Spot | May 26 – Aug 30 | May 24 – Aug 23 |
| Focal animal sampling | Macuco | May 2 – Aug 31 | May 15 – Sep 1 |
|  | Spot | May 17 – Aug 30 | May 9 – Aug 23 |

AD = adults (age > 6 yrs for males, > 5 yrs for females); SUB = subadults (age 5-6 yrs for males, 4-5 yrs for females); IMM = immatures (age 1 to 4 yrs for males; age 1 to 3 yrs for females). M = males, F = females. * Three adult males (BOR, HOR and PAC) entered the Macuco group for the first time in 2014; they were highly peripheral, and were not stable members of the group.

Table S3. Dates of treatment phases (Pre-Treatment = PRE-T and Post-Treatment = POST-T) according to the dates of administration of antiparasitic drugs to individuals from Macuco and Spot groups in 2013 and 2014. Since dates of drug administration varied among dewormed individuals within the same year and across the two study years, for untreated individuals, within each year and each group, we established dates of start and ending for each phase by calculating the percentage of individuals dewormed in each day, and assigning randomly an equal percentage of untreated individuals to each day. In red are indicated individuals who received the treatment and in black are reported Untreated (control) individuals who were assigned randomly to each date.

| **2013** | | |
| --- | --- | --- |
| **Macuco** | **PRE-T** | **POST-T** |
| Ofe, Crd, Maw | 13/5 - 16/6 (34 days) | 24/6 – 29/8 (66 days) |
| Ern, Srg, Tit, Edu, Sol, Srp, Mav | 9/5 - 17/6 (39 days) | 25/6 – 31/8 (67 days) |
| The, Est, Ric, Mgo, Yol | 2/5 - 27/6 (56 days) | 5/7 – 31/8 (57 days) |
| Ing, Fen | 9/5 - 1/7 (53 days) | 9/7 – 29/8 (51 days) |
|  |  |  |
| **Spot** |  |  |
| Jos, Eva, Tet, Tru, Rin, Bia, Dan | 17/5 - 26/6 (40 days) | 3/7-30/8 (58) |

| **2014** |  |  |
| --- | --- | --- |
| **Macuco** | **PRE-T** | **POST-T** |
| Crd, Ern, Maw, Ric, Sol  Ofe, Srg, Edu, Mav, The, Est, Hor, Bor, Ing | 15/5 - 15/7 (61 days) | 23/7 – 1/9 (40 days) |
|  |  |  |
| **Spot** |  |  |
| Bia, Dan, Jos | 9/5 - 15/7 (67 days) | 23/7 – 17/8 (25 days) |
| Rin, Tru, Eva, Tet, Rob | 9/5 - 17/7 (69 days) | 25/7 – 23/8 (29 da3ys) |

Red = Dewormed

Black = Untreated

Table S4. Model selection on the basis of Akaike Information Criterion (AIC), explaining variation in activity budgets for Foraging and Resting. †Global model, including the two experimental main factors (provisioning and antiparasitic treatment), and covariates such as year (2013, 2014), sex (males, females) and social rank (dominants, subordinates). The global model, the null model (including only experimental main factors) and the other competing models are provided. Models are listed in decreasing order of importance. For all models Individual ID and observation ID were included as random effects.

| **Response variables** | **Model** | **K** | **ΔAIC** | **AIC weight** |
| --- | --- | --- | --- | --- |
| FORAGING | Provis * Antipar + Year + Sex + Rank† | 9 | 0.00 | 0.871 |
|  | Provis + Antipar + Year + Sex + Rank | 8 | 4.46 | 0.094 |
|  | Provis + Antipar + Year + Sex | 7 | 7.51 | 0.020 |
|  | Provis + Antipar + Year + Rank | 7 | 8.19 | 0.015 |
|  | Provis + Antipar + Year | 6 | 15.68 | 0.000 |
|  | Provis + Antipar + Sex + Rank | 7 | 154.68 | 0.000 |
|  | Provis + Antipar + Sex | 6 | 156.59 | 0.000 |
|  | Provis + Antipar + Rank | 6 | 157.77 | 0.000 |
|  | Provis + Antipar (null) | 5 | 163.88 | 0.000 |
| RESTING | Provis + Antipar + Year + Sex + Rank | 8 | 0.00 | 0.663 |
|  | Provis * Antipar + Year + Sex + Rank† | 9 | 2.00 | 0.244 |
|  | Provis + Antipar + Year + Rank | 7 | 5.10 | 0.052 |
|  | Provis + Antipar + Year + Sex | 7 | 5.55 | 0.041 |
|  | Provis + Antipar + Year | 6 | 16.14 | 0.000 |
|  | Provis + Antipar + Sex + Rank | 7 | 19.17 | 0.000 |
|  | Provis + Antipar + Sex | 6 | 23.85 | 0.000 |
|  | Provis + Antipar (null) | 5 | 34.31 | 0.000 |
|  | Provis + Antipar + Rank | 6 | 2266.30 | 0.000 |

Table S5. Model selection on the basis of Akaike Information Criterion (AIC), explaining variation in individual centrality measures, i.e. Degree and Eigenvector centrality. †Global model, including the two experimental main factors (provisioning and antiparasitic treatment), and covariates sex (males, females) and social rank (dominants, subordinates). The global model, the null model (including only experimental main factors) and other competing models are provided. Models are listed in decreasing order of importance. For all models Individual ID was included as random effect.

| **Response variable** | **Model** | **K** | **ΔAIC** | **AIC weight** |
| --- | --- | --- | --- | --- |
| DEGREE | Provis * Antipar + Sex + Rank† | 8 | 0.00 | 0.945 |
|  | Provis + Antipar + Sex + Rank | 7 | 6.85 | 0.031 |
|  | Provis + Antipar + Sex | 6 | 8.50 | 0.013 |
|  | Provis + Antipar + Rank | 6 | 10.12 | 0.006 |
|  | Provis + Antipar (null) | 5 | 10.72 | 0.004 |
| EIGENVECTOR | Provis + Antipar + Sex + Rank | 7 | 0.00 | 0.423 |
|  | Provis + Antipar (null) | 5 | 1.29 | 0.222 |
|  | Provis + Antipar + Rank | 6 | 1.80 | 0.172 |
|  | Provis + Antipar + Sex | 6 | 2.46 | 0.124 |
|  | Provis * Antipar + Sex + Rank† | 8 | 3.95 | 0.059 |
